# Adaptive Mechanisms of Endemic Fish *Triplophysa yarkandensis* in the Tarim River Basin of Northwestern China

**DOI:** 10.3390/ani16203183

**Published:** 2026-10-10

**Authors:** Sadia Bibi, Yafan Zhu, Changcai Liu, Liting Yang, Kai Wang, Aiai Zhu, Liu Yu, Yong Song, Hongmei Li, Bin Huo, Shengao Chen

**Affiliations:** 1 College of Life Sciences and Technology, Key Laboratory of Tarim University, Tarim Research Center of Rare Fishes, Alar 843300, China; i107572025413@stumail.taru.edu.cn (S.B.); xygz48@hotmail.com (Y.Z.); 107572025416@stumail.taru.edu.cn (C.L.); 10757231127@stumail.taru.edu.cn (L.Y.); 10757251123@stumail.taru.edu.cn (K.W.); 10757252202@stumail.taru.edu.cn (A.Z.); 15223455435@163.com (L.Y.); 120050013@taru.edu.cn (Y.S.); 2 Shanghai Society of Oceanology and Limnology, East China Sea Ecology Center, Ministry of Natural Resources, Shanghai 200062, China; limailg@126.com; 3 College of Fisheries, Huazhong Agricultural University, Wuhan 430070, China

**Keywords:** endemic fishes, salinity–alkalinity stress, thermal tolerance, transcriptomic analyses, conservation biology

## Abstract

Dryland river ecosystems are among the most susceptible freshwater environments, with climate change, hydrological shifts, salinization and anthropogenic disturbance posing increasing threats to aquatic biodiversity. Endemic fishes in these regions are particularly vulnerable because of restricted geographic range, ecological specialization and narrow environmental tolerance limits, which reduce their capacity to cope with rapid environmental change. *Triplophysa yarkandensis* has a coordinated set of adaptive responses such as benthic morphological specialization, adaptable trophic modes, osmoregulatory ability to cope with saline–alkaline stress, and thermal tolerance mediated by stress-sensitive genes. Notably, muscle transcriptomic evidence indicates heat-associated expression changes in genes of the IRE1–TRAF2–MAP3K5–JNK signaling axis, suggesting this pathway as a candidate link between endoplasmic-reticulum stress and apoptosis-related responses. But its activation and causal role in apoptosis have not yet been confirmed at the protein and functional level. Nevertheless, these adaptations are increasingly constrained by habitat fragmentation, invasive species, declining water quality, and altered flow regimes, highlighting the limited buffering capacity of endemic species under increasing environmental disturbance. These observations provide a scientific basis for conservation planning, ecological monitoring, and adaptive management in arid inland waters, including habitat restoration, ecological flow regulation, and species-specific protection. Future studies must combine long-term field studies, multi-stressor experimental systems, and genomic–ecophysiological designs to define adaptive limits and improve conservation outcomes under continued climate change.

## Simple Summary

Freshwater fish living in dryland rivers face increasing threats from climate change, water shortages, increasing salt levels, habitat changes, and human activities. These challenges are especially serious for native fish species that survive only in limited areas. This study reviewed how the endemic fish *Triplophysa yarkandensis* adapts to the harsh environment of the Tarim River Basin and identified the factors affecting its survival. The review highlights key responses of *T. yarkandensis*, including osmoregulatory mechanisms that maintain ion balance under saline–alkaline stress, activation of the IRE1–JNK signaling axis associated with heat-stress-induced apoptosis, and the functional roles of the gut microbiota in metabolism, nutrient utilization, and stress resilience. The review also highlighted how changes in water availability, habitat loss, pollution, and introduced species may reduce its ability to survive in the future. Understanding these survival strategies provides important knowledge for protecting native fish populations and maintaining healthy freshwater ecosystems. The findings can support better conservation planning, habitat restoration, and management strategies to protect aquatic biodiversity in dryland river systems under future environmental changes.

## 1. Introduction

Drylands, which include arid and semi-arid regions covering nearly 40% of the global land area, are essential but highly climate-sensitive components of the Earth system [1,2]. Extreme climate change affects the pattern and frequency of precipitation, thereby threatening the security of sustainable water supply around the world [3]. River salinization and alkalinization are becoming important factors affecting the global river ecosystem [4], leading to the decline of biodiversity, the degradation of agricultural lands and freshwater areas, and the corrosion of infrastructure [5,6]. Water bodies, among the most anthropogenically transformed habitats, are now considered some of the most threatened ecosystems on Earth [7]. According to the Living Planet Report 2024, freshwater fish populations have declined by an average of 85% between 1970 and 2020, marking the steepest decline among terrestrial, freshwater, and marine ecosystems [8]. Climate change is expected to disproportionately affect endemic freshwater fish, which have limited distribution ranges, making them more vulnerable to environmental changes [9]. Similar declines have been documented in other arid-region river systems, including the Colorado River Basin (Southwestern USA), which has experienced a significant biotic restructuring, with severe declines in native mainstem fishes, including *Ptychocheilus lucius*, *Gila cypha*, and *Xyrauchen texanus*, all of these species have multiple populations that are now federally threatened, and several populations have been extirpated from the mainstem due to flow regulation, habitat fragmentation, and non-native species introductions [10]. Likewise, Australia Lake Eyre Basin has *Chlamydogobius eremius* [11] and the Murray–Darling Basin (Australia) has seen widespread declines in the distribution ranges of fish species such as *Maccullochella peelii*, *Bidyanus bidyanus*, and *Macquaria ambigua*, largely driven by changes in river flows and climate change impacts, and is considered to be experiencing high and persistent ecological risk in semi-arid river ecosystems [12].

In the arid northwestern region of China, water shortage has become the most severe limiting factor for socio-economic development and ecological stability. The Tarim River Basin, one of China’s most arid regions, is under especially intense water stress. Its fish fauna is extremely depauperate, with only 19 native species recorded, a number far lower than that of rivers of similar size elsewhere [13]. Endemic fish provide key insights into local adaptation due to their long-term evolution under specific environmental conditions; however, their narrow ecological tolerance and restricted distributions also make them highly vulnerable to disturbance and serve as effective indicators of freshwater ecosystem change [9]. The endemic fish species *Triplophysa yarkandensis* (Cypriniformes, Nemacheilidae), commonly known as Goutou fish (dog-head fish), is widely distributed across the Tarim River Basin network and plays an important ecological role [14,15,16]. Its restricted range, declining populations, and strong genetic structuring across the basin make it especially informative for understanding both adaptive responses and conservation needs in harsh inland waters [16]. Other endemic species, including *Triplophysa bombifrons*, *Schizothorax biddulphi*, and *Aspiorhynchus laticeps*, are endangered, underscoring the basin’s conservation importance [17,18]. This review mainly focuses on *Triplophysa yarkandensis* with the aim of synthesizing existing knowledge on its ecological adaptations, physiological and molecular mechanisms, environmental constraints, and conservation challenges undergoing environmental changes. This review is not intended to be a global overview of endemic fishes, but rather examples from other arid and semi-arid river systems are used as comparative references to help illustrate common ecological pressures such as habitat degradation, altered hydrological regimes and climate-related pressures and to provide broader insights for the conservation and management of *T. yarkandensis*.

## 2. Methodology

### 2.1. Literature Search Strategy and Study Identification

This structured literature search was conducted and reported in accordance with the ROSES guidelines for environmental evidence synthesis and the PRISMA 2020 reporting framework [19,20]. The authors performed the literature screening and selection by reviewing the titles, abstracts, and full text sequentially. The initial screening was performed by the first author, and ambiguous cases regarding eligibility were discussed among the authors until consensus was reached. This review was not registered as a systematic review, but as a structured evidence synthesis; therefore, no review protocol was registered in an international database. The search focused on the environmental adaptation, ecology, and conservation of *Triplophysa yarkandensis* or *Hedinichthys yarkandensis* in the Tarim River Basin. Both scientific names were included as search terms because *Hedinichthys yarkandensis* is recognized as the accepted name. In this study, *Triplophysa yarkandensis* is used as a synonym consistently throughout the study as a valid name according to Eschmeyer’s Catalog of Fishes and FishBase. The common names “Kashgarian loach” and “Yarkand loach,” together with the Chinese name “叶尔羌高原鳅,” were also used [21,22].

**Figure 1:**
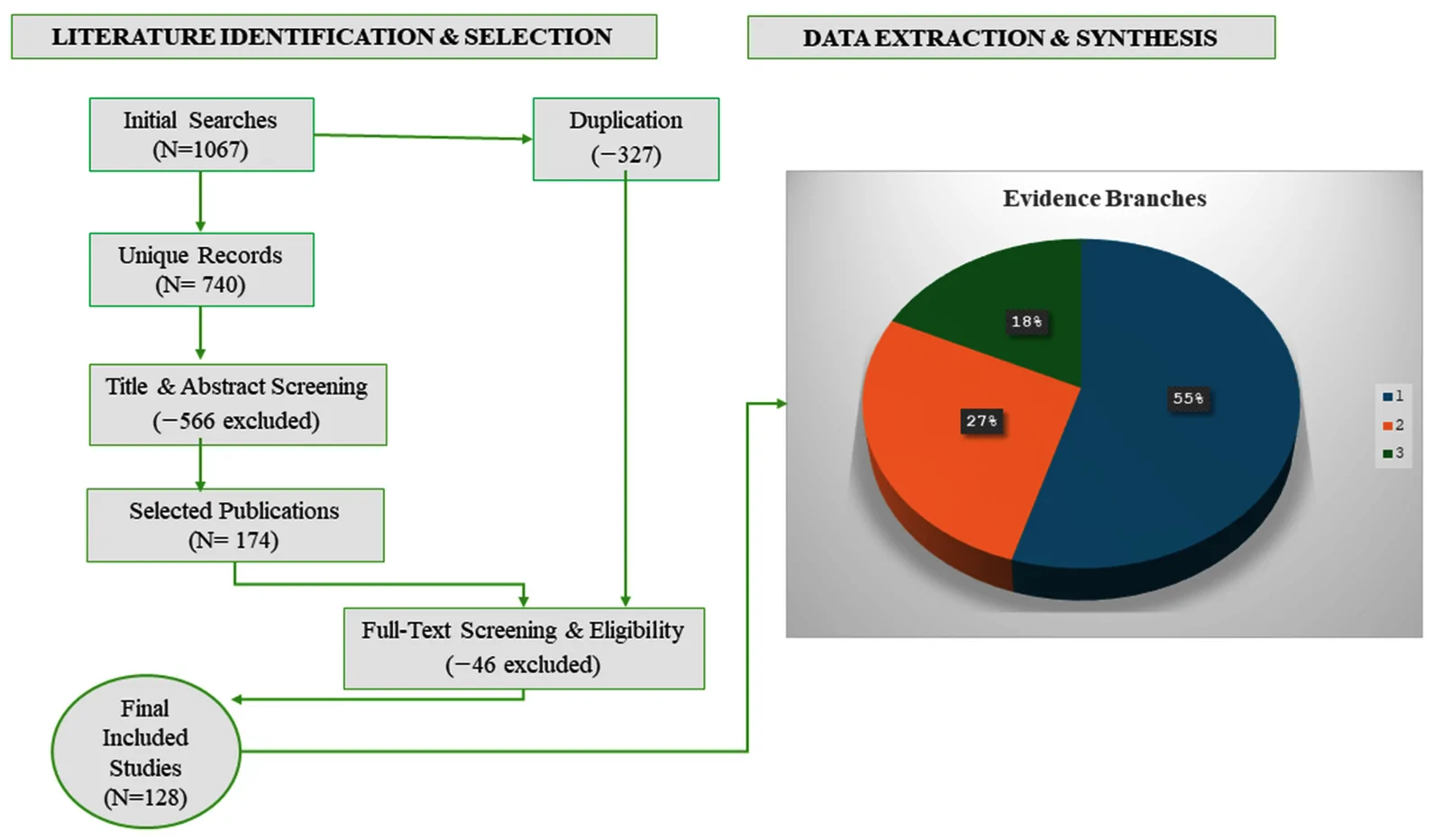
Systematic literature screening workflow following PRISMA/ROSES guidelines. From 1,067 initial records, 327 duplicates were removed, 566 were excluded during title screening, and 46 during full-text review, resulting in 128 eligible studies. Evidence was categorized into direct (1), supporting (2), and comparative branches (3).

The literature search was conducted between December 2025 and August 2026 using PubMed, Europe PMC, OpenAlex, and Crossref. Semantic Scholar was partially researched because of access and limitations. Supplementary searches were conducted using DOAJ, Google Scholar and directly on publisher websites, including ScienceDirect, Springer Nature, Wiley Online Library, Frontiers, and MDPI. The Chinese-language literature was identified through CNKI, Wanfang, CQVIP, and Baidu Scholar. The initial searches produced 1,067 records. Records were deduplicated using normalized DOIs, and when a DOI was unavailable, normalized article titles. Then we included the principal search expression combined with species, geographical and adaptation-related terms like (*Triplophysa yarkandensis* or *Hedinichthys yarkandensis* or Kashgarian loach or Yarkand loach), (Tarim River or Tarim Basin or Xinjiang), (adapt or physiology or saline or alkaline or thermal or heat or cold or drought or habitat or flow or growth or reproduction or genetic or transcript or microbiome or conservation, arid or dryland or desert or intermittent, and river or freshwater). Studies were excluded if they lacked relevance to adaptation or conservation for marine systems, other taxa, human health, archeological materials, aquaculture feed supplementation, or disease treatment or toxicology. This process removed 327 duplicates and left 740 unique records. Each of these publications was verified by visual inspection of the title and abstract, resulting in the selection of 174. Finally, 128 peer-reviewed articles were chosen after thorough text screening. Due to substantial variation in experimental designs, environmental exposures, biological endpoints, life stages, tissues studied, and study objectives among the included studies, a formal risk-of-bias assessment was not performed. Instead, the selected literature was categorized into three types of evidence: 70 (54.7%) studies were classified as direct evidence (studies specifically carried out on *T. yarkandensis*); 35 (27.3%) as supporting evidence (studies giving mechanistic or ecological background to the chosen case); and 23 (18.0%) as comparative evidence (studies available for related species or comparable environmental systems). This classification is to differentiate species-specific findings from general supporting evidence and to prevent extrapolation of evidence as direct evidence for *T. yarkandensis* (Figure 1).

Search phrases were modified to match the syntax of each database. There were no restrictions on publication years, and records in both English and Chinese were accepted. Primary studies were eligible if they: (i) contained empirical evidence on *T. yarkandensis*; (ii) examined another native fish species or ecological process in the Tarim River Basin with clear relevance to the conservation of the focal species; or (iii) examined fish adaptation in a comparable arid, desert, or intermittent freshwater system. Background information and taxonomic resources were not treated as primary sources of evidence but were retained for review and citation tracing.

### 2.2. Data Extraction, Evidence Categorization and Synthesis

**Figure 2:**
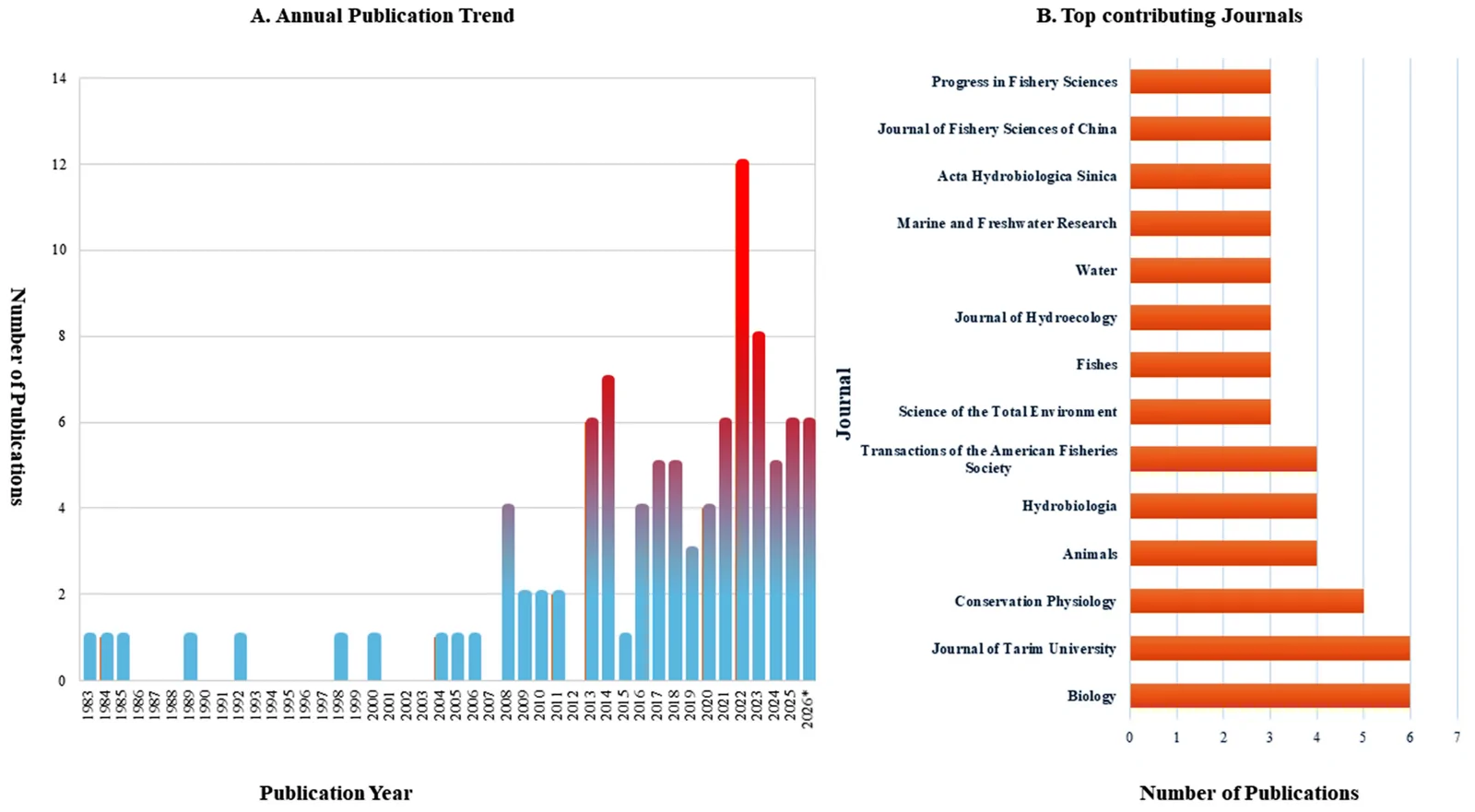
Bibliometric characteristics of the included literature dataset. (**A**) Annual publication trends of studies included in the review (1983–2026 *). * Indicates partial year through August 2026. (**B**) Number of publications among contributing journals.

The records were divided into three branches of evidence: direct evidence for *T. yarkandensis*, supporting evidence from other fishes in the Tarim River Basin, and comparative evidence from other arid freshwater systems. The information gathered from each potentially eligible study included bibliographic information, the geographic location of the study, focal taxon, life stage, sample size, study design, environmental driver, exposure intensity and duration, biological responses, main findings, reported limitations, and reported conservation relevance. If a study involved several species, populations, locations, or treatments, these were each treated as separate observations. The evidence was categorized into salinity–alkalinity tolerance, thermal and low-temperature responses, physiological energetics, growth and reproduction, genomic and transcriptomic mechanisms, population genetic diversity, microbiome variation, habitat fragmentation, flow alteration, and conservation management. The evidence from *T. yarkandensis* was the most interpretive. Evidence from the other fishes in the Tarim River Basin and from comparable arid-river systems was used to identify general adaptive mechanisms and research gaps. Descriptive data were used to summarize patterns in publication, study design, environmental drivers, and levels of biological response. The studies varied significantly in design, exposure, life stage, outcome, and reporting format, so effect sizes were not statistically pooled. Instead, the evidence was synthesized narratively following the guidance for synthesis without meta-analysis [23]. Inter-study differences were interpreted according to geographical location, life stage, tissue, magnitude of exposure, experimental duration, and the origin of the organisms (wild or cultured). In addition to evidence synthesis, descriptive bibliometric analysis was performed using the final dataset of 128 eligible studies to characterize temporal trends and research distribution. The annual publication output and contributing journals were summarized to illustrate the development of research interest in *Triplophysa yarkandensis* and related arid-river fish adaptation studies over time (Figure 2). The synthesis focused on examples of adaptive mechanisms and how they related to habitat protection, population connectivity, and long-term conservation.

## 3. The Tarim River and *Triplophysa yarkandensis*

**Figure 3:**
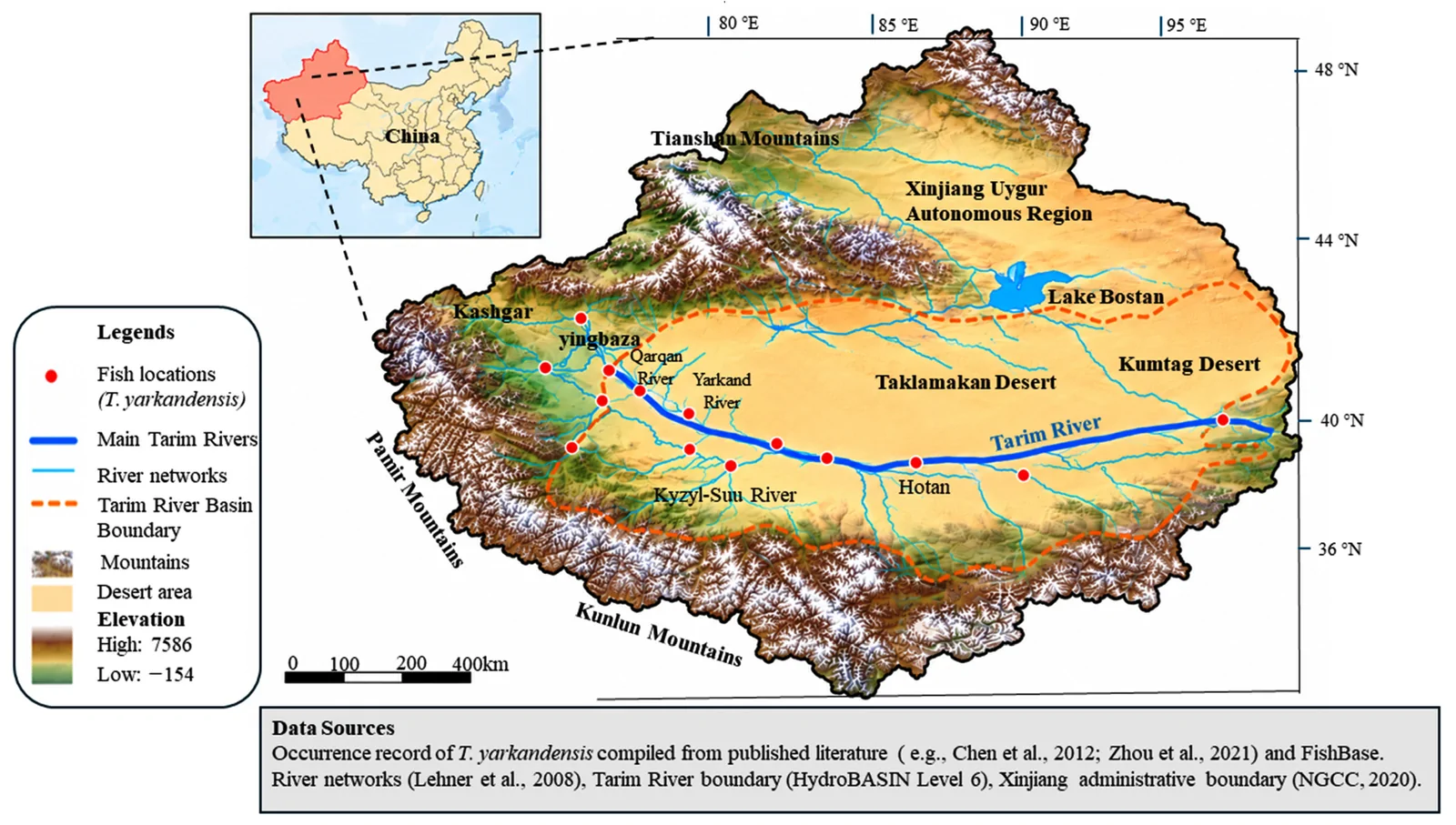
The distribution of *T. yarkandensis* is denoted by red circles across the Tarim River Basin. It highlights major rivers (blue) and mountain ranges (green), including the Tarim, Qarqan, Kyzyl-Suu, Hotan and Yarkand rivers. The map shows the geographic distribution of *T. yarkandensis* but does not demonstrate physiological tolerance for harsh climatic conditions [16,25,26,27,28].

Arid-region river basins worldwide, such as the Colorado River Basin and the Murray–Darling Basin, share similar environmental pressures, including water scarcity, salinization, and flow modification. These stressors have caused marked declines in native fish communities, particularly among endemic species with narrow ecological tolerances [10,12]. The Tarim River system in Xinjiang, China, harbors a depauperate but biogeographically distinctive native fish fauna [13]. *Triplophysa yarkandensis* is a benthic fish adapted to harsh conditions of dryland rivers, typically inhabiting slow-flowing shallow waters and favoring low-velocity marginal zones over main channels. It exhibits seasonally variable microhabitat use, with deeper water in the winter and nearshore benthos zones during other seasons [24]. The species is tolerant of cooler, saline–alkaline environments, and its habitat choice is influenced by hydrological and physicochemical factors [16] (Figure 3).

*Triplophysa yarkandensis* exhibits a carnivorous-leaning omnivorous diet, consuming mainly fish, aquatic insects, shrimp, and benthic invertebrates, with only minimal plant material [29]. Furthermore, its short intestine and its carnivorous diet suggest an adaptation for efficient exploitation of food resources in the benthic and near-benthic habitats. In the food webs of the Tarim River, the native species are thus likely a secondary or higher-level consumer that facilitates the transfer of energy from invertebrates and small fishes to higher trophic levels [30]. Reproduction primarily occurs during the warm season, specifically from March to August, with the highest spawning period occurring from April to June. In the Tarim River, gonadal development begins as temperatures reach approximately 20 °C in April, with increased flow and food availability; all these factors promote the development of ovaries, leading to peak spawning. Variable fecundity is a strategy that enables adaptation to high environmental instability in arid rivers [31]. Several other endemics in the region, such as *Diptychus maculatus*, *Aspiorhynchus laticeps*, *Schizothorax biddulphi*, and *S. irregularis*, are also highly specialized and considered at risk due to narrow distributions and sensitivity to environmental changes [32]. In nearby basins, species such as *Perca schrenkii* show similar adaptations to saline–alkaline conditions [33]. Golden perch (*Macquaria ambigua*) is a long-lived, highly mobile potamodromous generalist that uses hydrological cues like increased temperature, discharge, and flow variability to trigger spawning migrations to maintain connectivity, even within fragmented dryland river networks of the Murray–Darling Basin, Australia [34]. Stable isotope analysis is a powerful tool for assessing trophic niches and energy flow in aquatic ecosystems. In the Kizil Reservoir of the Tarim River Basin, δ^13^C and δ^15^N analyses showed that non-native fishes (*Silurus asotus* and *Ctenopharyngodon idellus*) occupied broader isotopic niches and strongly overlapped with native schizothoracines, including 98% niche overlap between *C. idellus* and *Schizothorax intermedius*. These findings suggest the existence of high dietary competition and resource sharing, which is consistent with the hypothesis that the successful invaders broaden their trophic niches, narrow the niches of the native species, and alter the flow of energy in food webs of arid inland rivers [35].

*Triplophysa yarkandensis* populations have declined due to multiple anthropogenic pressures; it has been recognized as a species of conservation concern and included in the list of key protected wild animals of the Xinjiang Uygur Autonomous Region [25,36]. It is an ecologically and economically important fish of high protein content, is locally popular for taste [37] and prolific breeding [31], but its population is becoming vulnerable due to climate change and other factors such as water diversion and habitat loss, which gives rise to serious concern for its conservation status [13].

## 4. Adaptive Strategies of *Triplophysa yarkandensis* and Other Arid-River Fishes

Climate change and anthropogenic activities are accelerating the salinization process in arid and semi-arid regions, where low rainfall, high evaporation, and water scarcity already create saline–alkaline conditions that impose severe osmotic stress on fish [4,38]. In arid inland basins, such as Northwestern China, saline alkali waters with high salinity, high pH, and complex ions cause high stress on fish and affect their survival, growth, and reproduction, driving strong adaptive selection. *Triplophysa yarkandensis* is an example of extreme adaptation in the Tarim River Basin, which has been optimized for feeding in low-quality turbid environments and reducing energy consumption through benthic morphology and specialized feeding strategies [30,39,40].

### 4.1. Morphological and Behavioral Adaptations

Behavioral flexibility in habitat selection enables dispersal-limited fish to be more resilient to long-term environmental changes [41]. Fish depend on habitats with essential environmental conditions, while shallow low-velocity zones are crucial for many native and threatened species, but these conditions are increasingly reduced in regulated river systems [42]. Fishes show habitat-linked movements for feeding, resting, and reproduction, and many potamodromous species exhibit multi-stage, size-dependent migrations throughout the year, with timing depending on the species and its specific life stage [43]. In floodplain rivers, endemic fishes typically show generalized feeding with low dietary overlap and rely mainly on rapid escape and inactivity for predator avoidance [44]. Under climate change, species are predicted to respond in three ways: adapt, shift range, or go extinct; species distribution models (SDMs) are used to predict these outcomes [45,46].

*T. yarkandensis* has a slightly elongate body with a cylindrical anterior portion, flat abdomen, laterally compressed posterior, and short caudal peduncle. Its head is short, thick, conical and dorsoventrally flattened, with a subterminal arcuate mouth, small eyes and three pairs of relatively long barbels [40]. The flattened head and abdomen likely enhance substrate contact, while fin placement and a reduced caudal region improve stability and station-holding in low-velocity benthic habitats. These morphological traits are accompanied by enhanced sensory adaptations. The barbels function as tactile and chemosensory organs, and the lateral line detects water flow and pressure changes, facilitating prey detection and orientation in low-visibility, turbid environments [39]. Experimental evidence from catfish confirms the lateral line function is critical for tracking prey in low-visibility conditions [47]. Murray cod use structurally complex habitats and flood-linked connectivity for movement, with about 58% dispersing from tagged waterholes and showing year-round activity, while regulated flows and water extraction disrupt recruitment cues and increase flood-related hypoxia, linking hydrological alteration to survival and population dynamics in the Murray–Darling Basin [48]. The razorback sucker (*Xyrauchen texanus*), the largest North American catostomid endemic to the Colorado River Basin [49], is adapted to variable, turbid flow regimes, with adults seasonally shifting habitats, preferring spring floodplain waters for foraging and thermal regulation [50]. In reservoirs, adults tend to roam within various microsites, but favor backwater areas and main impoundments. Larval razorbacks migrate from spawning waters to their nursery waters, the large backwaters [51], while reservoirs support movement among microsites, especially backwaters and impoundments. Larvae drift nocturnally from spawning sites into backwater nurseries that provide warmer, low-flow feeding environments [52,53]. Juvenile Colorado pikeminnow (*Ptychocheilus lucius*) rely on shallow, low-velocity backwaters and channel margins for refuge and foraging on abundant invertebrate prey. Natural floods and daily flow variations influence depth, velocity, and prey availability, creating dynamic but predictable refuges that influence habitat selection throughout the life stage [54].

The feeding apparatus of *T. yarkandensis* further suggests a benthic, bottom-oriented and opportunistic resource use. It has a short intestine (intestine-to-body-length ratio ≈ 1), which supports efficient digestion of animal-based food and a mouth structure adapted for capturing benthic prey and exploiting substrate-associated resources under changing conditions [30]. The species also shows adaptive external traits, including environment-dependent body coloration, smooth scaleless skin, and sexual dimorphism in pectoral fins, with males having stronger fins with elongated rays that may enhance benthic performance and camouflage [39]. The ecological habitat influences the adaptability of fish to their environment, and during reproduction, the habitat influences the ability of the larvae to exit the membrane and survive, which affects the survival rate of the offspring [55]. Gonadal hormones, water temperature, illumination, nutrition, water flow, and other factors influence gonadal maturity [29]. Overall, these traits support benthic stability, non-visual orientation, and efficient energy use in harsh, turbid, and hydrologically fluctuating inland waters (Table 1).

Other arid-region fish species also show coordinated morphological and behavioral adaptations that support survival in arid river systems. *Aspiorhynchus laticeps* has finely arranged silvery scales that streamline the body, reduce drag, and improve swimming efficiency, while its broad, flattened snout enhances sensory detection and gives it a predatory advantage. The broad and powerful caudal fin facilitates agility and evading predators, though its morphology and adaptive strategies are distinct [32], like fast-swimming fish Thunnus [56]. Additionally, its color-changing ability in response to the environment is a rare protective mechanism that aids in camouflage, reducing the risk of predation, akin to some coral reef fish, but more flexible and adaptable [32]. *Diptychus maculatus*, with its conical type of hypopharyngeal tooth, has evolved a more omnivorous feeding habit, which is suitable for cold climates. The long, coiled intestine allows for effective assimilation and absorption of plant fibers, a unique trait of phytophagous fish, while the lack of a stomach aligns with its omnivorous diet [32]. The Qinghai–Tibet Plateau offers an ideal rearing environment for *S. irregularis* and *D. maculatus* because of its cool water temperature, clear water visibility, and longer daylight hours, which are essential for their growth [57]. *Schizothorax biddulphi* has whiskers on its snout and jaws to sense food, and a horseshoe-shaped mouth to easily feed on algae from benthic substrates. Long, thin gill rakers enhance filter feeding by capturing phytoplankton and small aquatic insects, while reduced inner hypopharyngeal teeth efficiency supports this feeding mode. Hook-like pharyngeal teeth indicate an omnivorous diet, and an elongated, highly curved gut improves nutrient absorption, consistent with phytophagous and omnivorous habits [58]. These arid- and semi-arid-adapted fishes are benthic omnivores feeding on algae, insects, and detritus, overwintering in deep pools or gravel crevices and migrating upstream in spring for spawning in cooler waters [59].

**Table 1:** Phenotypic development of *T. yarkandensis* enhances survival through specialized morphology, sensory traits, and adaptable feeding behavior, maximizing the acquisition of resources.

Adaptation Category	Trait/Behavior	Adaptive Value	Reference(s)
Habitat-use adaptation	Benthic life in shallow, hydrologically variable waters	Favors persistence in unstable arid-river environments	[16]
Body morphology	Elongate body, cylindrical forebody, flat abdomen, compressed hindbody, short caudal peduncle, flattened conical head, subterminal mouth	Improves substrate contact, stability, and station-holding	[39]
Sensory morphology	Small eyes, long barbels, non-visual orientation	Enhances tactile/chemosensory detection and spatial orientation	[39]
Feeding adaptation	Omnivorous with carnivorous tendency; feeds on fish, insects, larvae, shrimp, and benthos; flexible benthic foraging	Supports opportunistic use of benthic food resources	[30]
Digestive adaptation	Short intestine; saline–alkaline digestive histological plasticity	Enables efficient digestion and epithelial protection under stress	[30,60]
External phenotype/coloration	Environment-dependent color change, spotted yellow-brown dorsum, pale ventrum, scaleless skin, modified male pectoral fin	May aid camouflage, benthic performance, and sex-specific function	[39]
Reproductive adaptation	Spring gonadal development linked to rising temperature, higher flow, greater food supply, and increased river volume	Synchronizes reproduction with favorable breeding conditions	[31]
Integrated adaptation	Coordinated morphological, sensory, trophic, digestive, and reproductive traits	Promotes benthic stability, efficient resource use, and reproductive success	[40]

### 4.2. Physiological and Osmotic Adaptations

Endemic fish in arid and intermittent rivers show strong physiological plasticity, allowing survival under drought, hypoxia, and habitat contraction. They maintain performance through adaptive oxygen transport and aerobic metabolism, including increased hematocrit, improved blood oxygen-carrying capacity, and preserved aerobic scope under low oxygen conditions [61,62]. Stress physiology, including energy mobilization and cortisol regulation, is central to survival in isolated remnant pools [63,64]. Golden perch exhibits high physiological plasticity and can survive and reproduce in variable riverine environments, including fragmented and ephemeral habitats. Juveniles quickly acclimate to hypoxia via improved oxygen transport and cardiovascular remodeling, while adults remain metabolically stable during hypoxia and low flow or temperature stress, allowing flood-timed spawning and movement between isolated pools [62]. *Maccullochella peelii* increases thermal tolerance via the upregulation of heat shock proteins, metabolic enzyme adjustments to sustain ATP production during periods of energetic stress, and improved gill ventilation and surface respiration during hypoxia [65]. Larvae and juveniles display size-dependent plasticity such that smaller individuals are more sensitive to hypoxia and larger juveniles to temperature, indicating ontogenetic allocation of metabolic capacity for stress adaptation [65,66]. Cardiovascular performance through heart rate and oxygen transport efficiency provides additional protection against temperature and oxygen fluctuations [67]. Adult *Ptychocheilus lucius* migrates 143 km and extends up to 200 km to spawn in oxygen-rich rapids and riffles, often outside its usual habitat range. Spawning during declining summer flows allows larvae to drift downstream into warm nursery areas that support rapid growth and reduced predation risk [68,69,70]. This spatial and temporal separation, including larval drift and adult feeding movements onto flooded banks, reduces intraspecific predation and enhances dispersal to productive nursery habitats [71]. The razorback sucker is adapted to the variable Colorado River Basin, with a bony keel for strong currents and flexible mouth and gill structures for feeding in turbid waters [72,73]. In general, females join males from nearby deeper water when they are ready for egg release. Adults are long-lived and migrate seasonally to spawn during spring high flows, while larvae drift into warm, sheltered backwaters for optimal growth and reduced predation [74,75]. Its broad habitat tolerance and flexible life-history strategy support long-term reproductive success, although river modification and introduced predators now threaten these adaptations.

**Figure 4:**
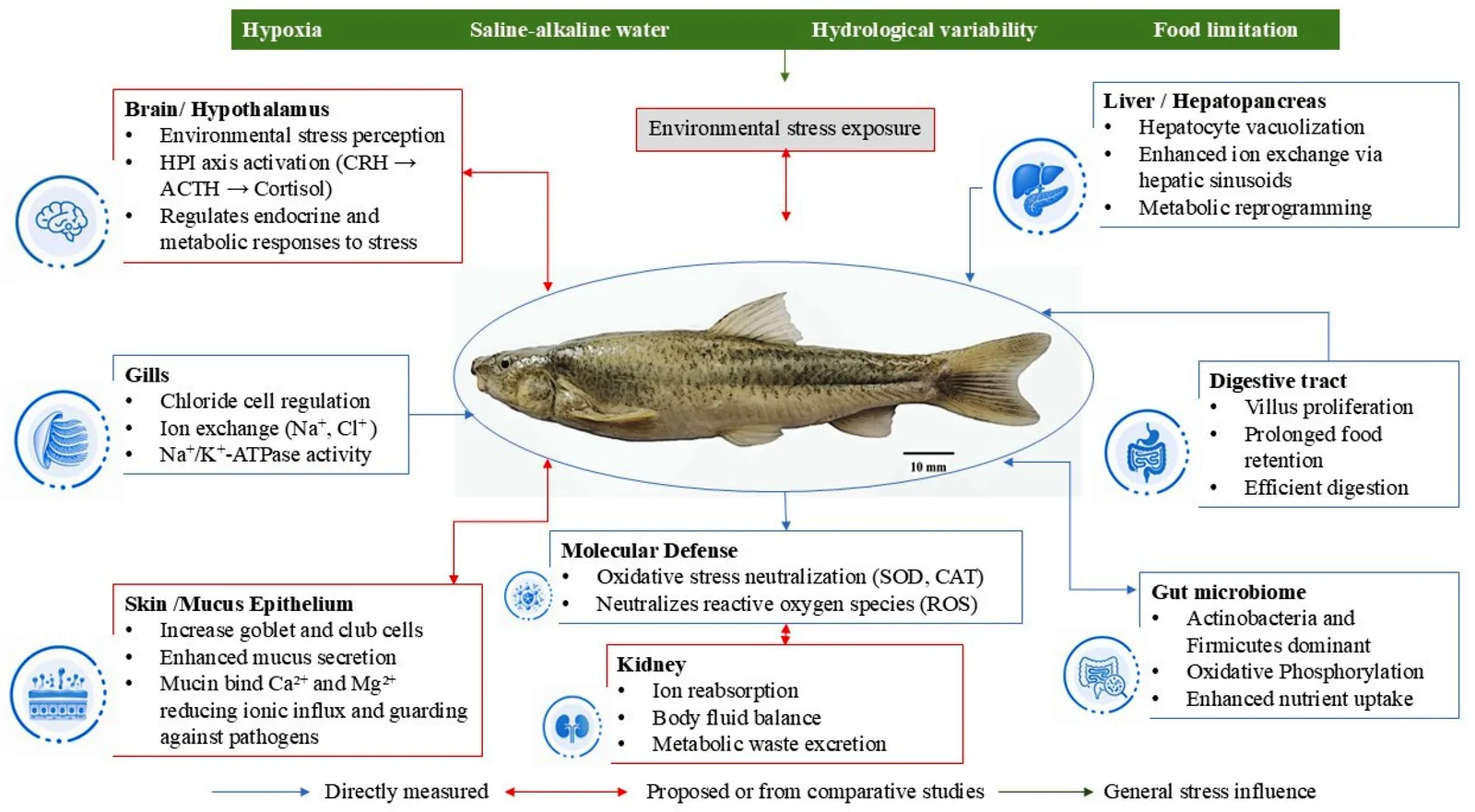
The physiological mechanisms of *Triplophysa yarkandensis* for survival in the harsh environment of the Tarim River Basin. Fish regulate stress hormones, ion exchange in the gills and mucus secretion to adapt to saline–alkaline conditions. The species also has metabolic and microbial adaptations for nutrient efficiency and antioxidant systems to combat oxidative stress. These strategies demonstrate the species’ adaptability and its potential for conservation in arid ecosystems.

Comparative salinity tolerance data for *T. yarkandensis* and other arid-zone fishes are summarized in Table 2. In *T. yarkandensis*, direct experimental evidence for saline–alkaline responses includes marked histological changes in the digestive system. Under saline–alkaline conditions, club and goblet cells and the number of intestinal villi increased, accompanied by some hepatopancreatic vacuolization compared with fish in freshwater controls. These changes reveal structural plasticity associated with mucus protection and digestive function [60]. Differential expression of genes related to ion transport, oxidative-stress responses, and metabolic pathways have also been directly observed in transcriptomic studies of fish exposed to saline–alkaline conditions [76], but these molecular changes alone do not establish the corresponding physiological mechanisms. Proposed mechanisms involving gill ion regulation, blood-ion control, organic osmolytes (e.g., betaine and inositol), and improved oxygen transport are thus treated as inferred mechanisms, not directly measured responses in *T. yarkandensis* (Figure 4). The mucus–epithelium–hepatopancreas model combines the secretion of mucus, the structural optimization of the epithelium, and metabolic remodeling in *T. yarkandensis*. Increased numbers of mucus-producing cells, proliferation of intestinal villi, and mild hepatopancreatic vacuolization have been directly observed under saline–alkaline conditions [60]. However, the proposed mucus–epithelium–hepatopancreas mechanism is partly generalized from other teleost fishes. Mucins are involved in the binding of Ca^2+^ and Mg^2+^, which decreases ionic influx through the esophageal epithelium and acts in synergy with the gill Na^+^/K^+^-ATPase to control osmotic pressure [77]. Gastric structural modifications increase food retention and food assimilation, which gives enzymes more time to act in an alkaline environment. The mild hepatocyte vacuolization and loosely arranged connective tissue between hepatic lobules promote stress adaptation and efficient ion exchange in hepatic sinusoids, whereas the increased level of antioxidant enzymes (SOD, CAT) help reduce oxidative stress, as observed in *Carassius auratus* [78], but these findings should be considered comparative evidence rather than direct evidence for *T. yarkandensis* [60]. In other euryhaline fishes, acute salinity changes can trigger temporary metabolic and physiological reactions such as cortisol, glucose and lactate changes and then acclimate with progression of time [79].

Shotgun metagenomic analysis identified differences in gut microbial composition and annotated gene content among *T. yarkandensis*, *D. maculatus* and *S. biddulphi* [80]. Actinobacteria in *D. maculatus* enhance survival under winter cold and starvation conditions. In *T. yarkandensis,* the presence of Thalassococcus and Kangiella was associated with its broad salinity ecology, although no experimental evidence of microbiome-mediated improvements in host salinity tolerance, metabolic efficiency or gut health was found. Firmicutes abundant in *S. biddulphi* and *T. yarkandensis* are associated with protein and carbohydrate degradation and may contribute to nutrient acquisition and metabolism. The gut microbiome of these species is consistent with an omnivorous to herbivorous feeding strategy. Correlation of specific bacteria to environmental tolerance should therefore be considered as hypotheses that must be validated by functional studies [80]. In Nile tilapia, a higher Firmicutes-to-Bacteroidetes (F/B) ratio has been associated with more efficient dietary energy and nutrient consumption but needs functional validation [81]. Overall, these patterns indicate that arid-river fishes use flexible regulation of respiration, metabolism, and stress responses to cope with highly variable, unstable environments.

**Table 2:** A comparison of experimental salinity exposure, tolerance, physiological, molecular, behavioral, and histological responses among *T. yarkandensis* and selected arid and euryhaline fishes. NR = not reported in the primary source.

Species	Life Stage	Exposure/Protocol	Duration/Acclimation	Salinity Exposure	Alkalinity	pH/Ionic Composition	Endpoint	Primary-Source Result	Reference
Arabian pupfish, *Aphanius dispar*	NR	Laboratory acclimation to five salinities	4 weeks at each salinity	<1, 35, 70, 105, 140 ppt	NR	NR	Physiological performance	Oxygen consumption, critical swimming speed, and routine activity were similar from <1–70 ppt but declined at 105–140 ppt. No LC_50_ was determined.	[82]
Arabian pupfish, *Aphanius dispar*	Adult	Direct transfer from near-freshwater desert-pond water to seawater	Fish held >8 months before experiment; sampled at 6 h, 24 h, 72 h, 7 d, and 21 d after transfer	1.92 ± 0.01 ppt → 42.68 ± 0.09 ppt	NR	pH 8.32 ± 0.03; seawater diluted with dechlorinated tap water for freshwater treatment	Molecular acclimation/survival	No mortality was recorded. Gill transcriptomic responses involved ion transport, osmoregulation, stress response, and tissue remodeling. Not an LC_50_ study.	[83]
Desert pupfish, *Cyprinodon macularius*	Juvenile	Na^+^-uptake kinetics after acclimation to dilute water	≥3 weeks acclimation to 2 or 7 mM Na^+^; flux exposure 0.9–2.3 h	Ambient Na^+^ 0.174–57.1 mM; preliminary 1 mM acclimation attempted	NR	Test medium: 480 μM CaSO_4_, 150 μM MgSO_4_, 100 μM KHCO_3_; pH 7.0; NaCl added	Na^+^ uptake; preliminary lower-acclimation limit	Attempts to acclimate fish to 1 mM Na^+^ caused ~50% mortality within 96 h. Main study quantified Na^+^ uptake kinetics; this is not a salinity LC_50_.	[84]
Desert goby, *Chlamydogobius eremius*	Adult male	Low- versus high-salinity behavioral treatment	~6→19 ppt over 24 h; ~4.8 d at 19 ppt; then target salinity reached over 24 h; 1-week final acclimation	5 versus 35 ppt	NR	NR	Reproductive behavior	Aggression and courtship occurred under both salinities; the study evaluated behavioral allocation under salinity stress. No lethal-tolerance endpoint was measured.	[11]
Amargosa pupfish, *Cyprinodon nevadensis amargosae*	NR	Rapid salinity change from baseline acclimation	7.5 ppt baseline; change completed over 4 h; sampled for 14 d	0.3, 35, or 55 ppt	NR	NR	Osmoregulatory/molecular response	Plasma osmolality and Cl^−^ rose after transfer to 35–55 ppt but returned toward baseline by day 14; gill Na^+^/K^+^-ATPase and ion-transporter expression changed during acclimation. Not an LC_50_ study.	[85]
*Triplophysa yarkandensis*	Adult	Slow-acclimation NaCl test	Fish held 1 week at 20 °C; 2 d experimental acclimation; salinity increased by 2 g L^−1^ d^−1^	Initial 0.30 g L^−1^; progressively increased NaCl	NR	Distilled water + NaCl; pH NR	LC_50_	LC_50_ = 16.51 g L^−1^ in spring. Distress occurred above 14 g L^−1^.	[38]
*T. yarkandensis*	Juvenile (~2 cm)	Slow-acclimation NaCl test	Same protocol as adult	Initial 0.30 g L^−1^; +2 g L^−1^ d^−1^	NR	Distilled water + NaCl; pH NR	LC_50_	LC_50_ = 12.94 g L^−1^ in spring.	[38]
*T. yarkandensis*	Larva (~0.6 cm)	Slow-acclimation NaCl test	Same protocol as adult	Initial 0.30 g L^−1^; +2 g L^−1^ d^−1^	NR	Distilled water + NaCl; pH NR	LC_50_	LC_50_ = 8.13 g L^−1^ in spring; larvae were the most sensitive life stage.	[38]
*T. yarkandensis*	Fish 8.5 ± 0.5 cm; 12.0 ± 1.0 g	Chronic saline–alkaline versus freshwater histological comparison	6 months	Saline–alkaline: 5.89 ± 0.32; freshwater: 0.325 ± 0.05	Saline–alkaline: 125.60 ± 25.12; freshwater: 25.12 ± 3.21; units reported as in source	pH stabilized with NaHCO_3_ buffer; numerical pH NR; saline–alkaline water characterized by elevated HCO_3_^−^/CO_3_^2−^	Histological response; not mortality	Increased club/goblet cells and intestinal villi, with mild hepatopancreatic vacuolization. 5.89 is an exposure salinity, not an LC_50_.	[60]
*Triplophysa strauchii*	Wild fish; 11 ± 0.53 g, 11.5 ± 0.61 cm	Acute NaCl salinity challenge	1-week laboratory acclimation; 96 h acute test	Control 0.645 ± 0.37 ppt; treatments 11, 11.7, 12.5, 13.3, 14.3, 15.1 ppt	NR	Acclimation pH 7.78 ± 0.09; analytical-grade NaCl used	96 h LC_50_/physiological response	96 h LC_50_ = 13.31 ppt; calculated safe concentration = 4.05 ppt; ≥14.3 ppt produced severe ion imbalance and physiological dysfunction.	[86]
Colorado pikeminnow, *Ptychocheilus lucius*	NR	Exposure to dilutions of naturally saline spring water	96 h	Natural saline spring-water dilutions; exact treatment series not available from publisher abstract	NR	Natural saline spring water from Glenwood Springs, Colorado; detailed chemistry NR	96 h LC_50_	96 h LC_50_ = 13.1 g L^−1^.	[87]

### 4.3. Thermal Tolerance and Stress Responses

**Figure 5:**
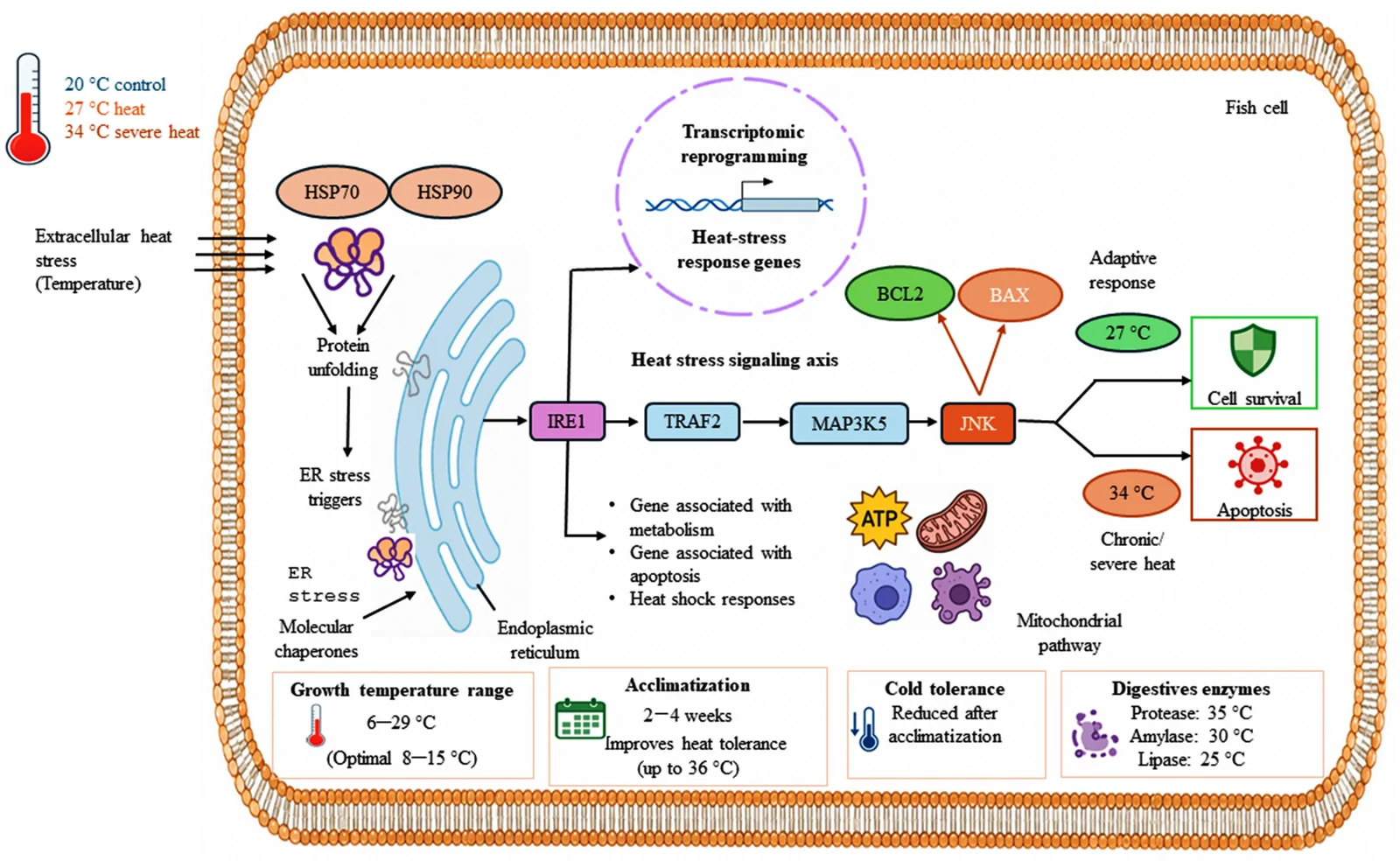
This illustrates the heat-stress response in *Triplophysa yarkandensis* to increasing water temperatures. It is activated by IRE1 and leads to activation of TRAF2, then MAP3K5, MAP2K7, and JNK, which control cellular heat-stress responses. The pathway has a balance between protective signaling BCL2 and apoptosis BAX, which depends on the concentration of stress. Short-term acclimation occurs between 6 and 29 °C, while temperatures above 36 °C trigger cell death, reflecting the species’ molecular adaptation to changing arid environments.

Thermal stress is a major factor affecting fish distribution and community structure, often leading to habitat loss and reduced reproduction in stenothermal species under warmer temperatures and extreme heat events [88]. Globally, approximately 25% of freshwater species are threatened by climate change, warming, habitat loss, and water extraction [89], particularly in climate-sensitive regions like the Tibetan Plateau [90]. Generally, cold-water species are less tolerant of heat than warm-water species. In the Tarim River Basin, warming combined with reduced water volume has weakened thermal regulation and contributed to the decline of fishery resources [36,91]. Fish are poikilotherms; temperature directly affects enzyme activity, feeding, growth, and metabolism, and prolonged heat stress can disrupt energy homeostasis and increase disease susceptibility [92]. In some fish species, thermal tolerance is body-mass dependent, with larger individuals generally showing lower heat tolerance but greater hypoxia resistance than smaller fish [65]. Murray cod may exhibit a lower, temperature-independent aerobic scope as an adaptation to the highly variable thermal climate of Southeastern Australia [67]. For Colorado pikeminnow, hatching and survival peak near 20 °C, with mortality increasing as temperatures fall below this threshold [93]. Razorback sucker tolerates a wide thermal range but prefers 18–24 °C, while larvae and juveniles show optimal growth in warm, shallow backwaters [73,94]. Other Tarim endemics show moderate temperature preferences. Juvenile *Schizothorax biddulphi* maintained >85% survival from 5 to 25 °C, with maximum growth at 20 °C [95]. *Diptychus maculatus* and *S. irregularis* show optimal growth at cooler temperatures (around 10–11 °C) and decline above 30 °C, indicating adaptation to cool, well-lit plateau waters [57] (Figure 5).

For *T. yarkandensis*, 6–29 °C has been reported as the growth range and 8–15 °C as an optimal growth range [96]. The heat-transcriptome experiment used 20 °C as its control and 27 °C and 34 °C as heat treatments [97]. These treatment temperatures do not independently establish growth optima and thermal limits. Nevertheless, the species showed improved heat tolerance after two to four weeks of acclimatization, with a maximum heat tolerance of 36 °C, whereas its cold tolerance was reduced [98]. The enzymes involved in digestion of *T. yarkandensis* also exhibit thermal adaptation; protease functions best at 35 °C, amylase at 30 °C in the stomach and intestines, and lipase at 25 °C in other body parts [99]. These results highlight the species’ tolerance mechanism, which is essential for its survival in the rapidly warming environment. However, the temperature range supporting optimal growth, successful reproduction, and long-term population resistance remain unclear.

### 4.4. Molecular and Genetic Bases of Adaptation

Genetic variation drives adaptation in arid and intermittent river fishes by maintaining adaptive capacity, supporting local physiological tolerance, and buffering populations against fragmentation-driven resilience loss, particularly in long-isolated Asian basins [100]. Schizothoracinae are cold-water fishes in which temperature changes with elevation, making temperature a key driver of genetic differentiation. In *D. maculatus* from Tashikuergan, the absence of lateral scales suggests strong selection under plateau conditions, potentially linked to low-oxygen adaptation where reduced scaling may enhance cutaneous respiration, like hypoxia-tolerant loaches. This species shows improved high-altitude adaptation, with genetic isolation closely associated with temperature variation. Overall, strong environmental gradients in temperature and altitude likely intensify divergent selection in *D. maculatus*, leading to reduced gene flow [101,102]. Population-level allelic variation in *Macquaria ambigua* is strongly correlated with environmental gradients that suggest local adaptation based on arid-region hydrology [103]. Under stress, fishes upregulate heat shock proteins, ion transporters, and metabolic enzymes to maintain homeostasis and improve thermal and osmotic tolerance, thereby enhancing their energy efficiency [76,97,104].

#### 4.4.1. Environmental Stress Signaling and Alkaline Adaptation

Environmental variability in arid river ecosystems imposes strong selective pressures on endemic fish species, requiring the integration of metabolic, cellular, and genetic regulatory mechanisms to maintain physiological stability. In Amur ide (*L. waleckii*), KEGG analysis of gill differentially expressed genes under alkaline stress identified EIF2 signaling, regulation of eIF4 and p70S6K signaling, and mTOR signaling as the top enriched pathways, highlighting their critical roles in stress tolerance. Genes governing transcription, metabolism, and cell communication were upregulated, whereas stimulus-genes were downregulated [105]. Liver enrichment pattern further indicated extensive metabolic reprogramming; oxidoreductase and transporter activities were upregulated, while transferase, electron carrier and other oxidoreductase activities were downregulated. Six oxidoreductase genes were differentially expressed between alkaline and freshwater. Strong regulation of ion and SLC-family transporters, including 128 gene accessions, reflected the high energetic demands of ion and organic-acid transport. Increased kidney proteolysis involved upregulation of aminotransferases, including tyrosine, aspartate, ornithine and alanine aminotransferases, while increased transferase activity of one-carbon unit, acyl group, glycosyl group, and phosphorus group transfer indicates a physiological response to environmental stress [105]. In *T. yarkandensis,* differential expression of *MAPK1*, *AQP1*, *ABCC1*, *CAT*, *GPX1*, and *GPX4* implicates pathways associated with antioxidant defense, ion transport, and osmoregulation without establishing evolutionary adaptations [76,106]. The oxidative responses to salinity may also vary with exposure time and acclimation state. For example, gill CAT, SOD, GPX, and MDA levels in *Acanthopagrus sheim* did not differ significantly among salinity treatments after 14 days, indicating that antioxidant responses to salinity are context-dependent [107].

#### 4.4.2. Thermal Stress Protection

In *Triplophysa yarkandensis*, transcriptomic studies reveal that multiple genes involved in pigment synthesis, metabolic regulation, stress signaling, and apoptosis pathways participate in the molecular response to environmental stressors such as temperature fluctuations, salinity stress, and oxidative imbalance. One of the early molecular responses to thermal stress appears to involve pigmentation metabolism. During tyrosine metabolism, differential expression of pigmentation-related genes such as *TYRP1* suggests possible involvement of melanogenesis-related processes in responses to environmental stress. This concept is supported by previous studies that show TYRP1 is involved in melanin deposition, providing protection against environmental stressors and cellular damage in fish tissues [97,108]. Thermal stress also triggers alterations in oxidative balance and metabolic regulation. The downregulation of *AOX* indicates altered aldehyde and redox metabolism under warm climate; however, AOX should not be interpreted as an antioxidant enzyme. Retinol metabolism, which is important for fish visual functions, also appears to be sensitive to environmental changes. The downregulation of *RDH* expression could reflect modulation of retinoid metabolism, potentially associated with visual physiology. However, a direct relationship between RDH expression and visual performance in *T. yarkandensis* has not been experimentally tested [109,110,111]. Similar mechanisms have been observed in mandarin fish, where RDH1 activity increases during feeding stimulation induced by artificial feed [112]. Transcriptomic evidence provides important clues regarding candidate pathways involved in environmental responses; however, gene-expression changes alone cannot establish functional consequences.

**Figure 6:**
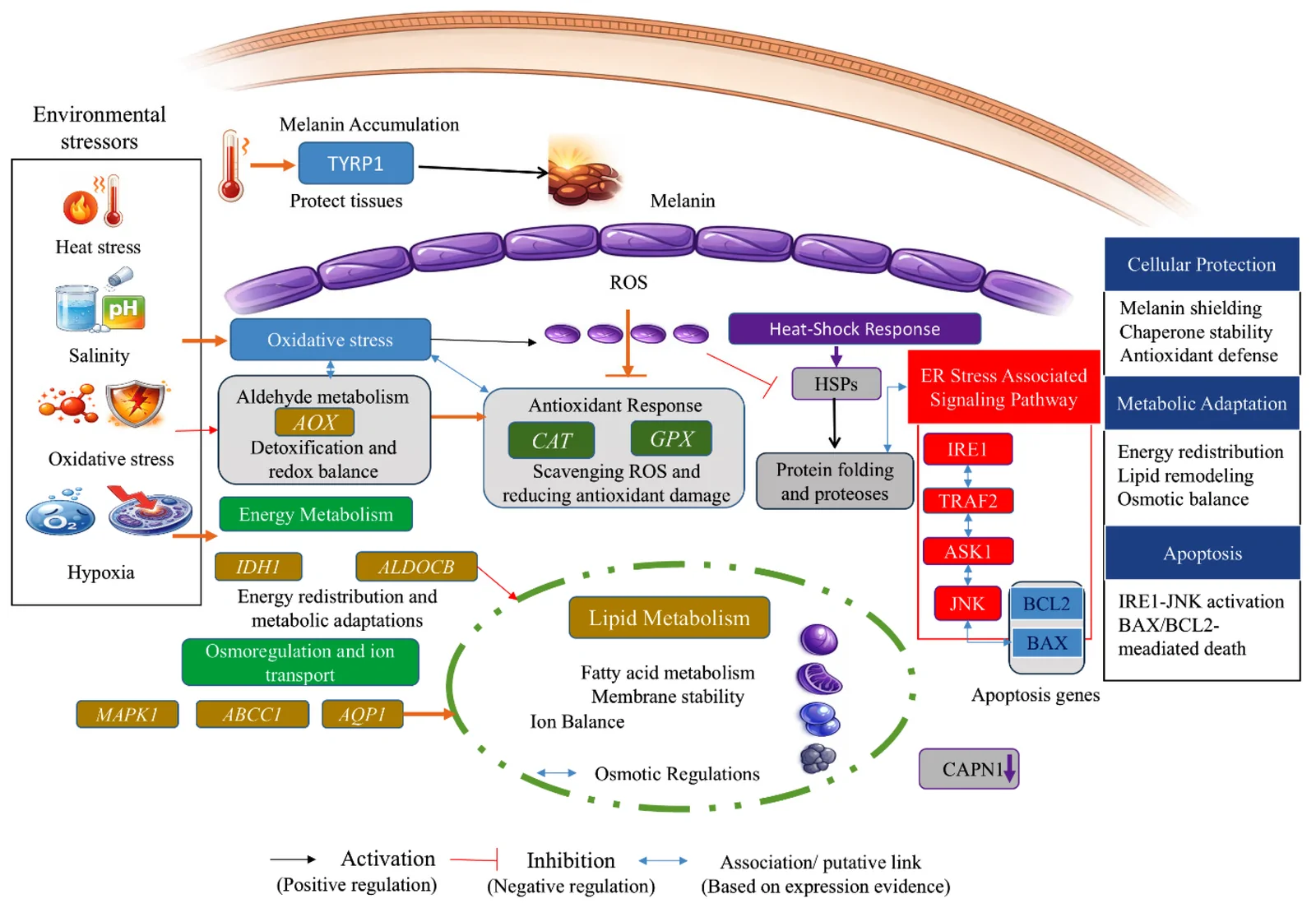
The coordinated molecular response of *Triplophysa yarkandensis* to environmental stressors highlights pathways that contribute to its survival in harsh conditions in the Tarim River Basin. The adaptive response begins with heat stress, salinity, and oxidative stress, followed by accumulation of melanin through the *TYRP1* gene, which protects the tissue from damage. When oxidative stress occurs, the pathway activates an antioxidative response, such as the activation of key enzymes including *AOX*, *CAT* and *GPX*, which are known to reduce oxidative stress. Additionally, energy metabolism is regulated through the expression of *IDH1* and *ALDOCB*, which enables energy metabolism to support life processes under stress. Lipid metabolism involving *MAPK1*, *ABCC1* and *AQP1* contributes to membrane stability and ion homeostasis, which is important for adaptation to saline–alkaline stress. The heat-stress response also stimulates heat shock proteins (HSPs) to maintain cell stability. The ER stress and apoptosis pathways, including IRE1, TRAF2, ASK1, JNK, BAX and BCL2, regulate cellular protection and apoptosis, determining whether cells survive or undergo apoptosis.

Heat shock proteins (HSPs) are highly conserved molecular chaperones that maintain protein homeostasis by helping protein binding, refolding, and avoiding accumulation. Their expression is regulated by heat shock transcription factors (HSFs), which bind to heat shock elements (HSEs) in HSP gene promoters [113,114]. They participate in protein synthesis, folding and assembly, translocation and aggregation control, primarily via HSP70 and HSP90 families, and are involved in the regulation of apoptosis, immune and inflammatory responses. Genes like HSPA1S, HSPA5, and HSP90A are highly upregulated under high temperature to maintain protein homeostasis, including protein refolding of misfolded proteins and protection against heat-induced damage [97,115,116]. As molecular chaperones, HSP70 proteins play roles in viral defense and autoimmune inflammation and are also important to maintain the integrity of cells under heat stress. In *L. waleckii*, heat stress significantly upregulates gill HSP genes, cathepsins and proteasome subunits under alkaline conditions. These systems are interconnected and are involved in the transport of damaged proteins to the proteasome for degradation to prevent toxic accumulation and the recycling of peptides and amino acids [105]. Stress signaling pathways regulate cell survival and metabolism during environmental stress. PI3K is a conserved lipid kinase family essential for membrane trafficking and signal transduction as second messengers in eukaryotic cells. Through its cytoplasmic pH domains, it recruits signaling proteins to the membrane to regulate cell growth, proliferation, differentiation, survival, and metabolism in response to environmental stimuli [117]. In *T. yarkandensis*, downregulation of *PIK3R1* suggests that physiological homeostasis is maintained during early warming, indicating stable immune function under moderate thermal stress (Figure 6).

The endoplasmic reticulum also contributes to cellular stress responses through protein processing. ER stress activates the unfolded protein response via activation of the PERK, ATF6, and IRE1 pathways to assist cell survival under adverse conditions, but also triggers apoptosis when stress is severe [118,119]. During heat stress, IRE1 activation enhances DNA damage and triggers apoptosis. In *T. yarkandensis*, higher temperatures significantly upregulate various genes related to ER stress, such as *IRE1*, *TRAF2*, *MAP3K5*, *MAP2K7*, *JNK*, *BAX*, and *BCL2*, and downregulate *CAPN1*. Transcriptomic analyses at 20, 27 and 34 °C have identified that activation of genes associated with the IRE1-TRAF2-MAP3K5-JNK signaling axis plays a key role in regulating apoptosis in the muscle of *T. yarkandensis* under heat stress [97]. In addition to stress-response signaling pathways, several genes associated with high-temperature tolerance have been identified in the muscle tissue of this species. These include heat shock proteins such as HSPA5, HSP70, HYOU1, and HSP40; ion transport-related genes such as *ATP2A*, *CACNA1A*, and *ATP1A*; and metabolic and regulatory genes including *TYRP1*, *ADH*, *WNT*, *CXCL14*, *CUL1*, *PKC*, *MTOR*, and *VCP*. Together, these genes form an integrated regulatory network that enables *T. yarkandensis* to cope with elevated temperature conditions. The IRE1–TRAF2–MAP3K5–JNK axis represents a proposed signaling mechanism linking endoplasmic-reticulum stress with cell-fate regulation. In response to moderate stress, the unfolded protein response supports protein homeostasis and ensures cell survival. When stress becomes severe or prolonged, IRE1-mediated recruitment of TRAF2 and activation of MAP3K5–JNK signaling can promote apoptosis, facilitating the elimination of severely damaged cells. This model thus correlates cell protection with damage elimination. *T. yarkandensis* also shows heat-induced expression alterations that confirm the potential role of this pathway; however, transcriptomic and qPCR data do not show protein activation, phosphorylation, or regulation of apoptosis that needs functional validation [97]. The identification of these genes provides an important theoretical basis for the conservation and artificial breeding of *T. yarkandensis* germplasm resources and contributes to the understanding of thermal adaptation mechanisms in other cold-water fish species [97].

#### 4.4.3. Energy, Carbohydrate, and Lipid Metabolism

Energy metabolism pathways play a critical role in maintaining cellular homeostasis under stressful conditions. In zebrafish, differential expression of ADH suggests changes in alcohol and aldehyde metabolic processes under stressful conditions, it does not demonstrate ethanol-producing pathways or diversion of pyruvate from lactate formation in fish species and would require enzyme or metabolite validation [120,121]. The enzyme encoded by IDH1 plays an essential role in maintaining intracellular energy metabolism balance, regulating lipid synthesis, and resisting oxidative stress. Previous studies have shown that carbohydrate metabolism genes such as *IDH1* and *ALDOCB* are upregulated under low-salt stress conditions, providing significant energy resources for fish species such as yellow drum (*Nibea albiflora*) to cope with environmental stress [122]. IPA pathway enrichment analysis revealed “Superpathway of cholesterol biosynthesis” in the liver, indicating that under severe environmental stress in the liver, the synthesis of cholesterol was significantly induced in the liver tissue of *L. waleckii* [105]. The results suggest that metabolic flexibility plays an important role in stress tolerance. In addition, fatty acid metabolism pathways also play a role in environmental adaptation in this species. Genes involved in lipid metabolism are highly expressed in the liver of T. yarkandensis, including *ELOVL4*, *ACACA*, *FASN*, *ACAA2*, *ACOX1*, and *ACOX3*. The upregulation of these genes suggests that *T. yarkandensis* has evolved mechanisms to maintain membrane stability and fluidity under low-temperature conditions. This is a crucial adaptation in this endemic species, as it enables lipid metabolic regulation to meet the challenges of cold-water environments and ensure cellular function and metabolic efficiency [76] (Figure 6).

#### 4.4.4. Population Genetic Structure and Demographic History

Population genetic studies have characterized genetic diversity and population structure in *T. yarkandensis*, providing information relevant to the conservation of its genetic resources [16]. Genome-wide SNP analysis revealed high genetic diversity and strong differentiation among nine populations in the Tarim River Basin, with an overall FST of 0.67. In contrast, mitochondrial cytb and D-loop analyses of eight populations revealed weaker differentiation, with 96.57% and 95.25% of genetic variation occurring within populations, respectively. Mitochondrial cytb and D-loop analyses show population structure and demographic history, including inferred historical changes in effective population size [123].

Heterogeneous study designs and exposure conditions limit comparisons across studies. Indirect and comparative evidence may not be directly applicable to *T. yarkandensis*, and proposed molecular mechanisms remain insufficiently validated experimentally. Physiological and transcriptomic changes thus provide evidence of environmental responses but do not establish causal mechanisms, fitness benefits or evolutionary adaptation.

## 5. Conservation Challenges and Strategies

Conservation of endemic fishes in the Tarim River requires an integrated, multi-scale approach that links environmental degradation to biological responses. Hydrological alteration and associated habitat fragmentation appear to be the primary threats to wild *T. yarkandensis* populations, followed by salinization–alkalinization and invasive species [16,106]. Dams and water infrastructure further isolate habitats and impede access to spawning and feeding areas, directly threatening species such as *T. yarkandensis* [16]. Dam-induced habitat degradation not only restricts migration, breeding, and feeding access, but can also reduce reproductive success, even though some fishes may adjust migratory behavior to maintain homeostasis under stress [124]. To conserve effectively, habitat quality must be improved, ecological connectivity preserved, and population resilience increased by improving habitat conditions, regulating flows, curtailing water withdrawals and controlling salinization in watershed management [125]. River connectivity should be maintained through fish passages and adaptive dam management to enable seasonal migration, as well as the protection of spawning, nursery, and refuge habitats critical for species like *Triplophysa yarkandensis* [31].

**Figure 7:**
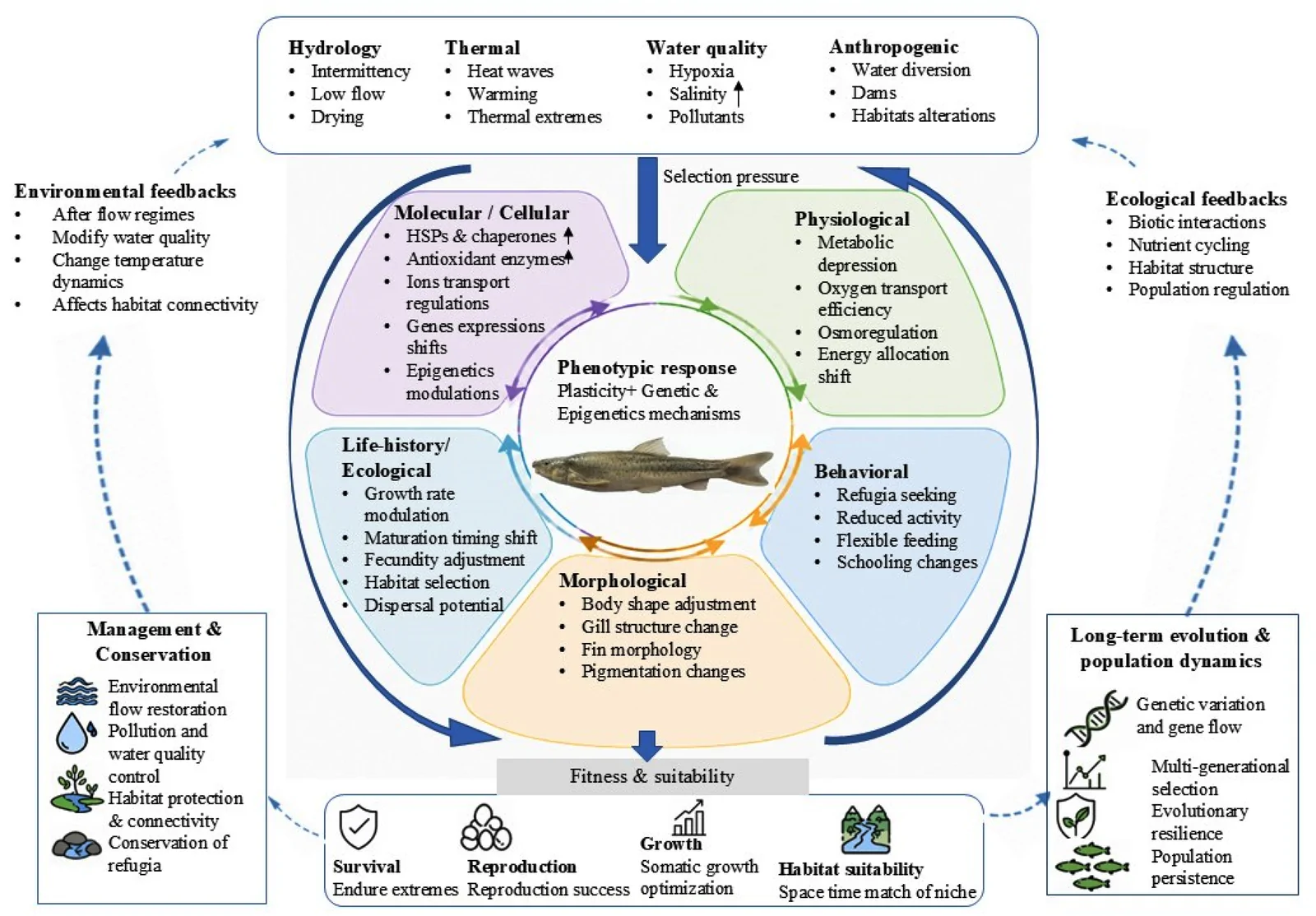
Demonstrates the adaptive mechanisms of *Triplophysa yarkandensis* to climate change and environmental stress. Hydrological changes, thermal extremes and anthropogenic water diversion act as environmental drivers that activate stress pathways (thermal, oxygen limitation, osmotic stress, and habitat fragmentation). This leads to physiological, molecular, morphological, and behavioral changes, such as metabolic depression, expression of heat shock proteins, changes in body shape, and refuge-seeking behavior.

Building on these physiological limits, we propose a staged ecological water-demand framework for conserving *T. yarkandensis* in the Tarim River that balances human water use, using its maximum thermal tolerance of 36 °C as a key constraint. In this framework, near-natural flows are maintained during the early season to protect spawning and juvenile habitats, ensure suitable water temperature and oxygen levels [126]. During peak irrigation periods, partial flows can be diverted to critical refugia to reduce thermal and oxygen stress while sustaining agricultural withdrawals. Multi-objective optimization suggests that such “designer flows” can maximize fish survival without significantly reducing irrigation capacity [127,128]. Once extraction is completed, flows should be restored to “near-natural” conditions based on physiological indicators such as temperature, dissolved oxygen, and stress biomarkers, thereby reconnecting fragmented habitats and promoting reproduction [129]. Tolerance depends on exposure duration and developmental stage, so it is important to have specific information on acute survival and chronic performance [130]. Temperature–oxygen interactions and body size can modify physiological responses, while salinity effects may depend on temperature and exposure conditions [131,132]. Reproductive stages deserve special attention as spawning adults and embryos may possess more limited thermal tolerances than other life stages [133]. These results are in favor of species-specific validation instead of direct transfer of thresholds to *T. yarkandensis*. These recommendations are partly supported by empirical evidence from the Tarim River Basin. Long-term ecological water conveyance has improved hydrological connectivity, groundwater conditions, and overall ecological quality in the basin, supporting the use of environmental-flow restoration [91]. The population genetic analysis of *T. yarkandensis* also shows significant spatial structure among populations, which highlights the need for habitat connectivity [16,134]. This integrated framework, linking environmental drivers, adaptive responses, and population resilience, is illustrated in Figure 7. Additionally, fish release programs can be used to restore populations of *Triplophysa yarkandensis*. Artificial breeding and release have been suggested as conservation tools for this species, and regional release programs are already implemented, but quantitative long-term evidence of the impacts of these activities on wild population recovery and genetic integrity is limited. For this reason, systematic field and genetic monitoring should also be part of the environmental-flow regulation and release programs [135].

Staged environmental-flow management is globally effective in sustaining riverine fish populations. The effectiveness of staged environmental flows is supported by international examples. Controlled flood releases in Australia’s Murray River have enhanced spawning and recruitment of native Percichthyids [136,137], and high flow releases from Glen Canyon Dam have restored backwater habitats for endangered Colorado River fish [138]. Such timed high-magnitude environmental flows can sustain fish populations while maintaining downstream water use [128,139]. As this staged ecological water-demand mechanism is mainly based on laboratory physiological parameters, before it can be applied for ecological water allocation in field-based operational management, its threshold values, stage boundaries, and effectiveness need to be validated. Combining multi-omics with ecological modeling further enhances conservation strategies. Genomic, transcriptomic and physiological data identify adaptive variation and primary stress-response pathways associated with ion regulation, oxidative defense, and hypoxia tolerance. Molecular signatures, when validated against functional traits such as metabolic rate and osmoregulation, can link stress response to fitness [76,140]. The integration of these data into ecophysiological and species distribution models allows prediction of habitat shifts, population risk, and climate refugia in climate change scenarios [141].

## 6. Conclusions and Future Directions

Endemic fishes of arid inland rivers are geographically restricted species that are survivors of long-term evolution in a harsh inland freshwater habitat. They persist under salinity, temperature fluctuations, hydrological stress and habitat fragmentation due to both natural selection and anthropogenic stress. *Triplophysa yarkandensis* exemplifies adaptation to such harsh conditions under increasing human impact. Histological and transcriptomic studies reveal tissue alterations and molecular responses to saline–alkaline and thermal exposure, and microbiome studies uncover the microbial composition and functional potential. However, these species are at great risk because of small niche breadth and vulnerable habitats, which restrict their ability to adapt quickly. Ongoing climate change, pollution, invasive species and increased water consumption pose a serious risk to their continued existence in dryland river systems. As a result, endemic arid-river fishes such as *T. yarkandensis* serve both as models of evolutionary adaptation and as sensitive indicators of ecosystem degradation. Future research should integrate multi-omics (metagenomics and metatranscriptomics) with longitudinal and infection challenge studies to elucidate gut microbiome functions, immune regulation, and genetic–epigenetic mechanisms underlying stress adaptation, reproduction, thermal tolerance, and growth in *T. yarkandensis*. Epigenetic regulation and transgenerational plasticity are two research directions that have not yet been investigated in *T. yarkandensis*. Results from studies of other fish taxa support further research on whether environmental stress can cause epigenetic alterations and whether associated phenotypic changes are transmitted across generations. These studies should differentiate between inherited effects from direct exposure and non-genetic parental influence. Experimental evolution, behavioral ecology and eco-genomic modeling should also be integrated to assess the potential for evolutionary rescue and to predict adaptive responses under climate change to give a comprehensive perspective on its resilience in extreme freshwater systems.

## Data Availability

No new data were created or analyzed in this study. Data sharing is not applicable to this article.

## Abbreviations

Following abbreviations are used during the preparations of this manuscript.

ADH	Alcohol dehydrogenase
ADH3	Alcohol dehydrogenase 3
AOX	Aldehyde oxidase
AQP1	Aquaporin 1
ATP	Adenosine triphosphate
ATP1A	ATPase Na^+^/K^+^ transporting subunit alpha
ATP2A	ATPase sarcoplasmic/endoplasmic reticulum Ca^2+^ transporting protein
BAX	BCL2-associated X protein (pro-apoptotic protein)
BCL2	B-cell lymphoma 2 (anti-apoptotic protein)
cytb	Cytochrome b
CAT	Catalase
CACNA1A	Calcium voltage-gated channel subunit alpha 1A
CAPN1	Calpain 1
CXCL14	C-X-C motif chemokine ligand 14
D-loop	Displacement loop (mitochondrial control region)
DNA	Deoxyribonucleic acid
EIF2	Eukaryotic translation initiation factor 2
ER	Endoplasmic reticulum
ELOVL4	Elongation of very long chain fatty acids protein 4
F/B ratio	Firmicutes-to-Bacteroidetes ratio
FASN	Fatty acid synthase
GPX	Glutathione peroxidase
GPX1	Glutathione peroxidase 1
GPX4	Glutathione peroxidase 4
HSPs	Heat shock proteins
HSP40	Heat shock protein 40
HSP70	Heat shock protein 70
HSP90	Heat shock protein 90
HSP90A	Heat shock protein 90 alpha
HSPA1S	Heat shock protein family A member 1S
HSPA5	Heat shock protein family A member 5
HYOU1	Hypoxia upregulated protein 1
IDH1	Isocitrate dehydrogenase 1
IRE1	Inositol-requiring enzyme 1
JNK	c-Jun N-terminal kinase
KEGG	Kyoto Encyclopedia of Genes and Genomes
multi-omics	Multiple omics approaches (genomics, transcriptomics, metabolomics)
MAP2K7	Mitogen-activated protein kinase kinase 7
MAP3K5	Mitogen-activated protein kinase kinase kinase 5
MAPK1	Mitogen-activated protein kinase 1
mTOR	Mechanistic target of rapamycin
MTOR	Mechanistic target of rapamycin gene
IPA	Ingenuity Pathway Analysis
PI3K	Phosphoinositide 3-kinase
PIK3R1	Phosphoinositide-3-kinase regulatory subunit 1
PKC	Protein Kinase C
RNA	Ribonucleic acid
RDH	Retinol dehydrogenase
RDH1	Retinol dehydrogenase 1
SLC	Solute carrier
SDMs	Species distribution models
SOD	Superoxide dismutase
TRAF2	TNF receptor-associated factor 2
TYRP1	Tyrosinase-related protein 1
VCP	Valosin-containing protein
WNT	Wingless-related integration site signaling pathway
WWF	World Wide Fund for Nature
